# Gene presence-absence variation associates with quantitative *Verticillium longisporum* disease resistance in *Brassica napus*

**DOI:** 10.1038/s41598-020-61228-3

**Published:** 2020-03-05

**Authors:** Iulian Gabur, Harmeet Singh Chawla, Daniel Teshome Lopisso, Andreas von Tiedemann, Rod J. Snowdon, Christian Obermeier

**Affiliations:** 1 Department of Plant Breeding, IFZ Research Centre for Biosystems, Land Use and Nutrition, Justus Liebig University Giessen, 35392 Giessen, Germany; 2 Section of General Plant Pathology and Crop Protection, Georg August University Göttingen, 37077 Göttingen, Germany; 3 College of Agriculture and Veterinary Medicine, Jimma University, Jimma, Ethiopia

**Keywords:** Plant breeding, Polyploidy

## Abstract

Although copy number variation (CNV) and presence-absence variation (PAV) have been discovered in selected gene families in most crop species, the global prevalence of these polymorphisms in most complex genomes is still unclear and their influence on quantitatively inherited agronomic traits is still largely unknown. Here we analyze the association of gene PAV with resistance of oilseed rape (*Brassica napus*) against the important fungal pathogen *Verticillium longisporum*, as an example for a complex, quantitative disease resistance in the strongly rearranged genome of a recent allopolyploid crop species. Using Single Nucleotide absence Polymorphism (SNaP) markers to efficiently trace PAV in breeding populations, we significantly increased the resolution of loci influencing *V. longisporum* resistance in biparental and multi-parental mapping populations. Gene PAV, assayed by resequencing mapping parents, was observed in 23–51% of the genes within confidence intervals of quantitative trait loci (QTL) for *V. longisporum* resistance, and high-priority candidate genes identified within QTL were all affected by PAV. The results demonstrate the prominent role of gene PAV in determining agronomic traits, suggesting that this important class of polymorphism should be exploited more systematically in future plant breeding.

## Introduction

Duplication of genes followed by diversification is a common process shaping the evolution of plant species by natural and artificial (breeding) selection^1^. Genes can be duplicated by different mechanisms, including tandem duplication, transposon-mediated duplication, segmental duplication, or in the most extreme form by whole-genome duplication (WGD) or polyploidization. WGD is common in the evolutionary history of many wild and cultivated plant species. Different terms have been used frequently to describe short- and long-range genomic duplication and genome structural variation (SV), a term originally defined in reference to insertions, deletions and inversions greater than 1 kb in size^2–4^. In contrast to small-scale insertion-deletion (InDel) polymorphisms, which are generally defined as small insertions or deletions of a few nucleotides (up to 50 bp), SV in the size range of genes (up to a few kb) can give rise to copy number variation (CNV) or presence/absence variation (PAV). The latter is an extreme form of CNV where fragments in the size range of genes are missing from the genomes of some investigated genotypes.

Genes affected by duplications, InDels, CNVs and PAVs in diploid and polyploid plant species have been linked to local adaptation of wild populations^5^ and to important agronomical traits in crops^1,3,6–8^ for example flowering time and vernalization requirement in oilseed rape^9,10^ and wheat^11^, abiotic stress tolerance in wheat^12,13^ and biotic stress tolerance in tobacco^14^. However, the strong impact of CNV and other forms of SV in polyploid crop genomes on evolution and trait selection was not recognized until the last few years, when recognition of their relevance was facilitated by large-scale genotyping including long-range sequencing technologies in large breeding populations of numerous crops^15^. One recent genotyping technology which is particularly suitable for detection of long-range SV is Bionano optical genome mapping using nano-channel arrays^16^. This method involves imaging of high-molecular weight, fluorescently-labeled DNA molecules and creation of large restriction maps represented as stretches of light and dark regions (resembling a barcode), which then can be aligned to an *in silico* generated optical map of a reference genome assembly. A key factor distinguishing this approach from previous technologies for SV analysis is that the DNA molecules are not sheared, thus enabling the capture of long-range genomic information stretching up to several hundred kilobases. In combination with accurate genome assemblies even for strongly complex polyploid crop genomes, optical mapping opens new avenues for dissection of genomic rearrangements associated with traits relevant for commercial plant breeding.

Oilseed rape (*Brassica napus*) is a recent polyploid crop species originating from the inter-specific hybridization between the two diploid progenitor species, *B. oleracea* and *B. rapa*. Due to high levels of homoeology between the two progenitor subgenomes, widespread structural rearrangements are a common phenomenon within the rapeseed genome^17–19^, while its ancestral hexaploid progenitor genomes already carried intensive structural and functional modifications through long-term genome fractionation and evolution^20–23^.

This study focuses exclusively on gene PAV in oilseed rape breeding populations and evaluates the biological relevance and prevalence of gene PAV associated with resistance to a common fungal disease of oilseed rape, Verticillium stem striping caused by *Verticillium longisporum*. By genetic mapping of genome-wide SNP/SNaP markers^24^, along with short-read Illumina sequencing in combination with long-range optical mapping, we demonstrate that inclusion of presence-absence polymorphisms in quantitative trait locus (QTL) mapping strategies enables also reliable identification of gene PAV with a putative role in *V. longisporum* resistance. The results provide a valuable example for the importance of pangenomic gene variation for breeding of a key trait in a major polyploid crop.

## Results

### QTL detection for *V. longisporum* resistance

**Figure 1:**
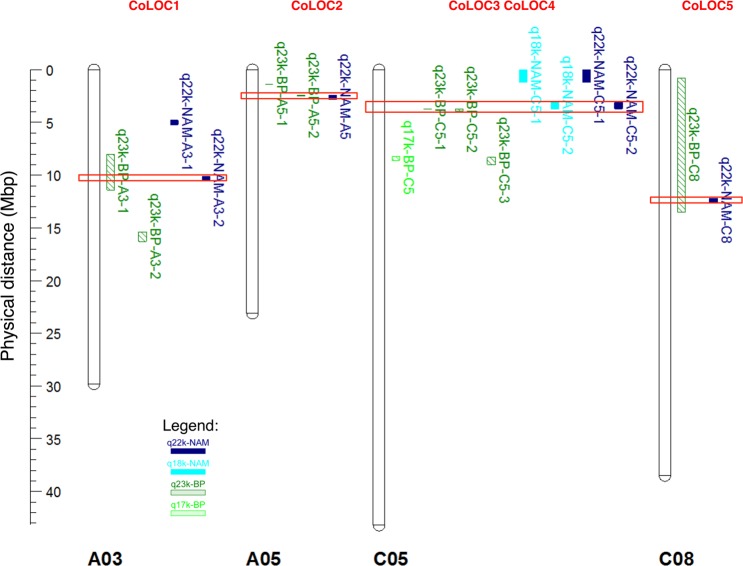
Comparison of co-localizing (CoLOC) QTL positions (in Mbp anchored to Darmor-*bzh*) obtained by QTL mapping in the biparental ExR53-DH population using maps produced with SNP markers only (light green, hatched) or SNP plus SNaP markers (dark green, hatched), and by GWAS in a NAM panel with 5 subpopulations using maps produced with SNP markers only (light blue, solid) or SNP plus SNaP markers (dark blue, solid). Only QTL above a threshold or LOD > 3 and −log (p-value) > 3 were included in the figure. Red boxes indicate QTL regions overlapping in biparental QTL and GWAS.

Analysis of raw genotype data from the Brassica 60k SNP array^25,26^ for a doubled haploid population ExR53-DH was performed for 244 genotypes. Two genetic maps were produced, one using only SNP markers (the “SNP map”) and one using SNP plus SNaP markers^24^ (the “SNaP map”), respectively. Comparison of the SNP and SNaP maps revealed that large chromosomal regions were not covered in the SNP map (e.g. for chromosome A03 compare Fig. 1a,b). Surprisingly, QTL mapping using the SNaP map increased the number of detectable QTL from 5 to 17 (Supplementary Table S1), with substantially increased LOD scores also indicating a dramatic increase in QTL detection power when including SNaP marker data. Furthermore, the map resolution and precision across QTL intervals were considerably increased by inclusion of SNaP markers. Interestingly, some QTL detected only in the SNaP map contained only SNP markers within the QTL confidence interval (e.g. q23k-BP-A1-1, q23k-BP-A3-2, q23k-BP-A3-2, q23k-BP-A5-1), while other QTL spanned intervals containing only SNaP markers (e.g. q23k-BP-C2-1). Many SNaP markers clustered in groups, spanning large regions up to chromosome scale, while other SNaP markers were located within blocks of SNP markers (Supplementary Table S1).

Additionally, a subset of subpopulations from crosses of a common elite oilseed rape parent with five synthetic *B. napus* parents was selected based on segregation of parental lines for *V. longisporum* resistance from the *B. napus* nested association mapping (NAM) panel described by Snowdon *et al*.^27^. GWAS including both SNP and SNaP markers increased QTL detection power and detected a total of 41 significant marker-trait associations (Supplementary Fig. S2, Supplementary Table S2). Most of the additional detected QTL harboured only SNaP markers.

### Co-localizing resistance QTL in diverse genetic backgrounds

Using only SNP markers, comparison of QTL detected by biparental QTL mapping and by GWAS revealed no co-localizing QTL (Fig. 1 in light green and light blue, Supplementary Tables S1 and S2). In contrast, adding SNaP markers to the QTL analyses revealed a strong increase to 5 co-localizing QTL (Fig. 1 in dark green and dark blue, Table 1) harbouring 2 to 90 genes per confidence interval (Supplementary Table S3). Sizes of QTL intervals for *V. longisporum* resistance showed differences between the biparental QTL mapping and the multi-parental GWAS (Supplementary Tables S1 and S2). Generally, smaller QTL intervals are expected in a NAM-GWAS approach, as higher numbers of recombinations are expected from crosses involving multiple non-related parents. On the other hand, QTL intervals in a GWAS mapping approach can only be measured for markers which can be positionally anchored, whereas biparental mapping can also consider marker loci which can be genetically, but not physically anchored. This led to some co-localizing resistance QTL with smaller confidence intervals observed in the biparental mapping compared to the NAM-GWAS (e.g. QTL on chromosomes A05 and C05, Table 1).

**Table 1:** Comparison of QTL locations detected by biparental QTL mapping in ExR53-DH and by GWAS in a NAM panel using SNP and SNaP markers.

chromosome	QTL ID in biparental population	Start position of QTL interval in ExR53-DH	Stop position of QTL in ExR53-DH	Size of QTL interval (bp)	QTL ID in NAM population	Start position of QTL LD block in NAM population	Stop position of QTL LD block in NAM population	Size of LD block (bp)
chrA03	q23k-BP-A3-1	7,963,059	11,419,476	3,456,417	q22k-NAM-A3-2	10,075,388	10,458,202	382,814
chrA05	q23k-BP-A5-2	2,357,535	2,473,365	115,830	q22k-NAM-A5	2,384,153	2,808,636	424,483
chrC05	q23k-BP-C5-1 and q23k-BP-C5-2	3,670,200 and 3,688,115	3,672,842 and 3,949,617	2,642 and 261,502	q22k-NAM-C5-2	3,089,132	3,698,279	609,147
chrC08	q23k-BP-C8	801,925	13,488,675	12,686,750	q22k-NAM-C8	12,201,749	12,596,542	394,793

### Gene ontology and enrichment analysis for genes underlying resistance QTL

Gene ontology enrichment analysis of the biparental population revealed five enriched GO terms in 17 QTL regions, with highest significance (topgoFisher score) attributable to the terms ‘chitin catabolic process’ and ‘response to biotic stimulus’ (Supplementary Table S4). In the NAM population eight enriched terms for genes harboured in 28 QTL regions were mainly related to cell growth. In the co-localizing QTL sections six enriched GO terms were identified. A total of 144 genes are harboured within the confidence intervals of the 5 co-localizing resistance QTL (CoLOC1 to CoLOC5, Supplementary Table S5). The GO terms for the 144 genes harboured in the 5 co-localizing QTL intervals reflect annotations from just 8 genes (labelled in green in Supplementary Table S5) related to cell-wall modification (expansins) and pathogen defence (defensins) on chromosome A05 (CoLOC2) and selenium binding on chromosome C08 (CoLOC5). One gene each returned annotation terms containing ‘response to stress’ and ‘systemic acquired resistance’, respectively. For four genes an annotation term ‘defense response to fungus’ or ‘response to symbiotic fungus’ was assigned, mostly based on plant defensin genes, whereas seven genes returned annotations containing the term ‘cell wall’, mostly based on expansin genes and pectin esterase-like protein genes.

### Both long and short-range PAV associate with *V. longisporum* resistance

To validate if consecutively mapped SNaP markers can help to reliably detect deletions within gene-range size associated with otherwise invisible QTL, resistance-associated QTL detected in the ExR53-DH population were physically located in the *B. napus* Darmor-*bzh* reference genome and the corresponding sequences were compared with optical genome maps. *De novo* assembly for parental lines Express 617 and R53 was performed using 300 Gb (~250x coverage) from Express 617 and 140 Gb (~116x coverage) of Bionano data, respectively. DNA molecules from both genotypes exhibited a very high N50 > 180 kbp, ensuring that long-range genomic information was covered (Supplementary Table S6). One nick label was detected for every 10,000 bp of the molecules, also indicating a uniform coverage and a high SV detection power. The final assembly comprised 1,368 and 1,331 optical maps with N50 values of 234 kb and 235 kb, respectively. The total lengths of the optical mapping assemblies were 978 Mb for Express617 and 874 Mb for R53. The high molecule sizes, along with the total size of the assemblies close to the predicted genome size for *B. napus*, indicate a good assembly quality suitable for reliable detection of long-range SV. Large-scale deletions were consistently detected by consecutively anchored SNaP markers in the ExR53-DH genetic map as well as by optical mapping data (Supplementary Fig. S1). Optical maps enabled accurate detection of small to medium-size deletions and insertions in the size range of genes (from 3 to 5 kb).

The 17 detected QTL regions harboured between 3 and 21 SNP and/or SNaP markers. In total, 122 markers were contained within QTL regions and 72 were anchored to the Darmor-*bzh* reference genome (Supplementary Table S7). All regions were investigated for long-range structural variants within QTL regions in parental genomes by analyzing the optical map data and comparing the data with SNP/SNaP marker patterns in the segregating population. Short to medium -range structural variation (deletion <5 kb) were confirmed for 16 out of 18 (89%) SNaP marker positions in the two parents (Supplementary Table S7), suggesting that genetically anchored consecutive SNaP markers can reliably detect short to medium-range presence/absence polymorphism associated with *V. longisporum* resistance.

**Figure 2:**
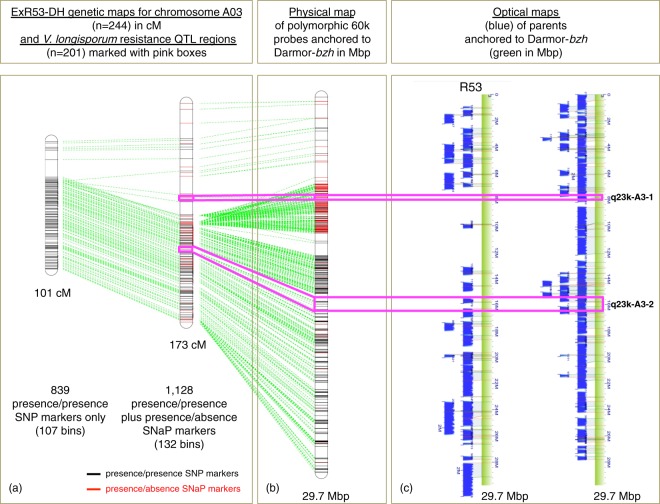
Genetic and physical localisation of biallelic SNP and presence/absence SNaP markers in two *V. longisporum* resistance QTL on chromosome A03 in the ExR53-DH population and its parents. (**a**) Genetic linkage maps showing positions of biallelic SNP (black) and SNaP (red) markers. Green lines connect consensus markers between the different map versions. (**b**) Positions of SNP probe sequences anchored by BLASTn to the Darmor-*bzh* reference sequence. (**c**) Optical Bionano genome maps (blue) of the two parental lines Express617 and R53 aligned to the Darmor-*bzh* reference sequence (green). The pink lines connect marker positions flanking QTL regions in the SNaP map, the physical map and the optical maps (**c**). No resistance QTL were detected using the genetic SNP map.

Figure 2 shows an example for comparison of genetic mapping from the DH population, reference anchoring of SNP and SNaP markers and optical mapping data of two parents for chromosome A03, which harbours two additional QTL not detectable using the SNP map. One of the QTL detectable only with the SNaP map, q23k-BP-A3-1, harboured 4 SNaP markers, whereas another, QTL q23k-BP-A3-2, harboured 14 SNP markers within the confidence interval (Supplementary Table S7). The QTL q23-BP-A3-1 is localized in a region with long consecutively ordered stretches of SNaP markers. For the parental line Express 617, both QTL regions were covered in the optical maps (Fig. 2c), whereas for the synthetic *B. napus* parent R53 about 6 Mb overlapping the QTL region q-23k-BP-A3-1 was not covered. The region not covered in R53 corresponds to a 13.8 cM interval on the genetic SNaP map (3.456 Mb), with the resistance allele contributed by Express 617 (Fig. 2a,b). This lack of optical map alignments in QTL q23k-BP-A3-1, combined with segregating, consecutively anchored SNaP markers in the segregating DH population, confirms the deletion of this large chromosomal region in parental line R53 and suggests that this deletion is involved in resistance expression. In contrast, the second QTL, q23k-BP-A3-2, is located in a region on chromosome A03 with long consecutively mapped stretches of SNP markers. Flanking the QTL region, only isolated SNaP markers were mapped. The isolated SNaP markers detected close to the QTL thus probably represent short-range PAV, potentially down to even single-nucleotide level. Nevertheless, saturating the genetic map on chromosome A03 by adding SNaP markers facilitated the detection of QTL q23k-BP-A3-2, whereas no QTL could be mapped in this region using only the SNP map (Fig. 2a, Supplementary Table S1). This example demonstrates that the addition of SNaP markers for genetic mapping was causal for a higher detection power of *V. longisporum* resistance QTL associated with both presence/presence as well as presence/absence polymorphisms.

### Gene PAV is a key determinant of *V. longisporum* resistance

In order to identify putatively absent genes from regions associated with *V. longisporum* disease resistance in the NAM panel, we combined Illumina 60 K SNP chip array genotyping and Illumina resequencing data for the six NAM parental lines with GWAS data from the segregating NAM population. Illumina resequencing confirmed medium to long-range presence/absence variation in the respective parents for 17 QTL regions (Supplementary Table S6). 60% of the 28 detected resistance QTL were affected by medium to long-range PAV in the NAM population.

**Figure 3:**
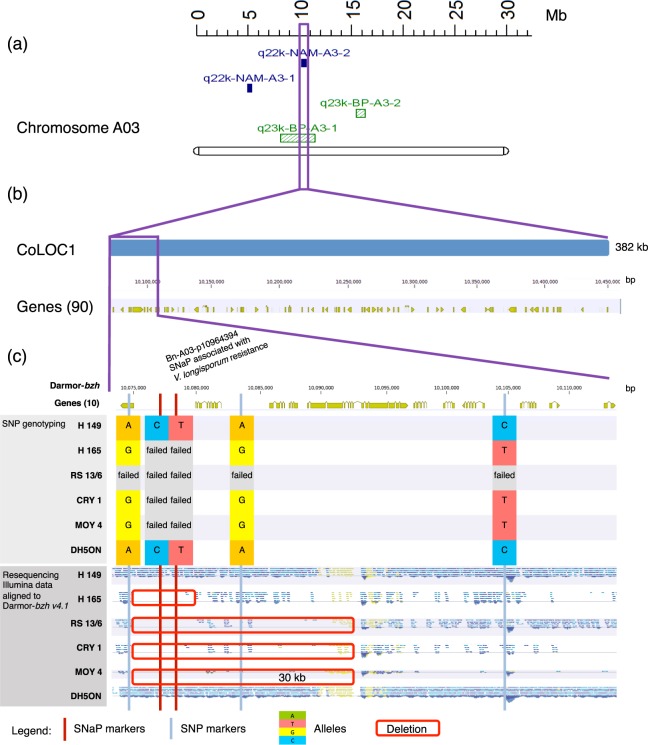
Comparison of SNP/SNaP marker polymorphism, sequence coverage and gene content at the co-localizing QTL CoLOC1 on *B. napus* chromosome A03. (**a**) Positions of QTL for *V. longisporum* resistance in the NAM panel (purple) and in the biparental mapping population ExR53-DH (green). (**b**) Chromosome interval and annotated genes in the QTL region in the *B. napus* Darmor-*bzh* v4.1 reference genome. (**c**) Allele patterns of reference-anchored markers in parents of six NAM subpopulations along with resequencing coverage data in the six parents. Red boxes indicate confirmed segmental deletions involving genes. Failed alleles represent SNaP (absence) alleles in susceptible parents.

Figure 3 shows an example of the analyses for the SNaP marker Bn-A03-p10964394, which is associated with *V. longisporum* resistance within the QTL interval q22k-NAM-A3-2 (Supplementary Table S8). This region CoLOC1 (Supplementary Table S3) also overlaps with QTL q23k-BP-A3-1 detected in the biparental population (Fig. 3a). Comparison of the marker-trait segregation in the NAM panel with marker and resequencing data for the six parental genotypes confirmed the expected pattern and revealed a putatively deleted interval within a part (8%) of the QTL region of the susceptible parent. This deletion corresponds to a 30 kb region containing 4 protein-coding genes (Fig. 3c, red box) within the entire QTL interval of 382 kb containing a total of 90 genes (Fig. 3b). However, from 5 markers (3 SNaPs, 2 SNPs) within the LD block of the co-localizing QTL interval CoLOC1, only the SNaP marker Bn-A03-p10964394 within the deleted region in R53 is significantly associated with *V. longisporum* resistance, suggesting that presence/absence polymorphism is involved in resistance. Based on the resequencing data of the parental lines, two to four genes are affected by deletions within the QTL interval (Fig. 3c), namely BnaA03g21190D (coding for an uncharacterized protein), BnaA03g21200D (coding for a skp1-like protein involved in ubiquitin-dependent protein catabolic processes), BnaA03g21210D (coding for aquaporin pip1-2 protein involved in transporter activities), BnaA03g21220D (coding for an uncharacterized protein) and BnaA03g21230D (coding for an ATP-dependent helicase brm-like protein involved in DNA binding).

SNP/SNaP marker patterns within *V. longisporum* QTL regions were also compared with parental whole-genome sequence coverage in the two parents of the biparental population ExR53-DH. Complete or partial presence/absence events were reconfirmed by resequencing coverage analysis of the two parents for 12 out of 18 reference-anchored SNaP markers (67%) and for 5 out of 6 QTL regions harbouring one or several reference-anchored SNaP markers (83%, Supplementary Table S9). The 2.5 cM QTL interval q23k-BP-A3-1 spans 3,45 Mb on the Darmor-*bzh* reference, containing 683 genes (Supplementary Table S9). Part of this region is shown in detail in Fig. 2 as co-localizing QTL region CoLOC1. Within CoLOC1 a total of 382 kb, containing 90 genes, overlaps in the biparental and multi-parental QTL analyses (see above, Supplementary Table S3 and Fig. 3).

Using genomic resequencing data from 52 accessions^28^, including the six NAM parents investigated in our study, we tested the software package SGSGeneLoss^29^ for gene loss calling and classification of genes as PAV genes or non-PAV genes. Parameters of 5% minimum gene size coverage (lostCutoff = 0.05) and minimum coverage of two reads for a gene (minCov = 2) was used by Hurgobin *et al*.^19^ to call a gene as present in a genotype. However, using these parameters for the 6 NAM parents used in our study, and comparing gene loss calling with PCR amplification data for 11 gene fragments from two genomic regions on chromosome A03 and C04 (Gabur *et al*.^24^, Fig. 3 and Supplementary Fig. S3) revealed an inaccurate gene fragment loss classification in 9 of 66 cases (86% accuracy) and no PAV was called for the chromosome C04 regions. This suggests that the chosen parameters were not stringent enough in the six genotypes to distinguish genuine gene loss from data processing noise due to misalignments. Hence, gene loss was accordingly underestimated using these SGSGeneLoss program parameters (and using Darmor-*bzh* v.4.1 as a reference). We therefore developed a customized pipeline which calibrated gene loss calling parameters based on PCR wet lab data and also included the gene size in the calculations to reduce further alignment biases. This procedure increased accuracy of gene loss calling to 96%. Both methods were then used for gene loss calling in the six NAM parents, Express617 and R53, firstly across the whole genome and then within identified QTL regions. Using our modified approach, 49% of total genes (49,701 of 101,039) were estimated to be affected by gene loss and 51% of genes within the 28 QTL regions (1,334 of 2,601) were estimated to be affected by gene loss (PAV genes) between the 6 NAM parents. Between Express 617 and R53, 23% of total genes (23,772 of 101,039) and 23% of genes within 17 QTL regions (590 of 2,646) were estimated to be affected by gene loss. A total of 144 genes were harboured in the 5 QTL intervals which co-localized between the biparental and multi-parental QTL mapping. Of these, 74 (52%) in the NAM panel and 37 (26%) in the ExR53-DH panel were estimated to be affected by PAVs. The genes estimated to be affected by PAV overlap in 20% of cases within the 5 co-localizing QTL regions between the two populations (Supplementary Table S10).

For all genes including PAV genes (144) within the co-localizing QTL regions, 30% of genes have no annotation (43 out of 144). This could suggest that some of the gene models are bioinformatics artefacts. However, from the 43 not annotated genes, 38 (92%) had expression in pan-transcriptome data from He *et al*.^30^ or in RNASeq data from the ExR53-DH population (mock or *V. longisporum*-infected, Supplementary Table S11). This suggests that the lack of gene annotation does not necessarily mean that the gene models are incorrect. 12% of all gene models in Darmor-*bzh* could not be annotated using Blast2GO. This is in contrast to the 30% of genes we found in the co-localizing QTL regions which could not be annotated using Blast2GO. Thus, the prevalence of these transcript-validated genes in QTL regions which cannot be annotated from public databases seems to be not a random phenomenon.

## Discussion

The reanalysis and integration of SNP array data, short-range Illumina sequencing data and long-range Bionano optical mapping data with QTL data provided new insights into the importance of gene PAV for disease resistance expression against Verticillium stem striping in oilseed rape. QTL analyses including presence/absence markers increased the power of detection for *V. longisporum* resistance in two *B. napus* breeding population. This type of variation (genic or nongenic) was previously reported to be associated with quantitative resistance against two other major fungal disease of oilseed rape 24 suggesting that it is a common and prevalent phenomenon in *B. napus*.

The recent allopolyploid crop species *B. napus* shows a strong abundance of SV and genomic rearrangements^18,31^. In maize, Beló *et al*.^32^ used comparative genomic hybridization arrays for detailed genome-wide analysis of SV, and a more recent study discovered some degree of CNV between 100 analyzed lines across more than 90% of the maize genome^33^. However, to date it is unknown how widespread this phenomenon is in other, older polyploid crop species and it is unclear for *B. napus* and most crops how many genes, what kind of genes and what traits are predominantly affected. Particularly for complex polyploid crops, considerably higher numbers of diverse, high-quality reference assemblies are required before reliable pangenomic analysis of genome-wide gene PAV becomes possible. Hurgobin *et al*.^19^ classified 38% of all *B. napus* genes in a diversity set of 53 genotypes as PAV-affected, including genes involved in important agronomical traits. In the NAM parental lines, Express617 an R53 we classified 49% and 23% of all genes as PAV-affected by using a PCR-calibrated gene loss bioinformatics pipeline. As five of the 6 NAM parents are resynthesized from exotic *Brassica* species a high gene loss could be expected which are known to exhibit an elevated ratio of rearrangements and SVs^17,18^. Surprisingly the percentage of genes affected by PAV was similar within the whole genome, the *V. longisporum* QTL for both populations suggesting that the prevalence of gene PAV is a general phenomenon randomly distributed throughout the genome and not restricted to *V. longisporum* resistance or other traits. For gene cluster, gene families and LRR genes involved in qualitative disease resistance it has documented that gene PAV is multiple times higher than on average within the entire genome^19^. However, this seems not to apply for quantitative resistance where diverse genes involved in complex pathways influence resistance responses.

Different mixtures of *V. longisporum* lineages/pathotypes were used for disease resistance screening in the two different *B. napus* mapping populations. Comparison of QTL regions detected by including SNaP markers between the biparental and the multi-parental populations revealed a considerable number of common QTL. This indicates broad-spectrum, lineage/pathotype-independent resistance reactions in genetically diverse germplasm which are of great interest for commercial resistance breeding. Thus we focused for a more detailed analysis on these 5 co-localizing QTL regions, which harbour 2 to 90 genes each within the co-localizing section of the QTL intervals. Based on the quantitative genetic nature of the disease resistance, we assumed that single specialized genes involved in very different biological functions from each QTL interval will not contribute to *V. longisporum* resistance, but rather a number of genes from common biosynthesis pathways or with common biological functions from one or all QTL intervals. To prioritize candidate PAV genes putatively involved in broad-range resistance expression we performed a gene ontology enrichment analysis for all QTL-associated genes, separately for both populations, and also for the genes from the 5 colocalizing QTL intervals. The enriched GO terms for the genes from the QTL regions were quite different between the two populations, being mainly related to chitin and cell-wall metabolism for the biparental ExR53-DH population and mainly related to cell growth for the multi-parental NAM population. This difference is not unexpected, as the disease resistance screenings are known to be highly susceptible to the environment and slightly different mixes of *V. longisporum* lineages/pathotypes were used for inoculation of the two populations. However, the result might also indicate that common as well as different resistance mechanisms are activated by different pathotypes.

Within the 144 genes from the co-localizing QTL putatively involved in pathotype-independent resistance, we found a number of genes coding for selenium-binding proteins, for plant defensin proteins and for expansin proteins have been found to be affected by PAV. In *A. thaliana*, expression of selenium binding proteins is tightly linked to detoxification processes related to oxidative stress^34^. Plant defensins are major components of the innate immune system of plants, are involved in the cell wall integrity signaling pathway and often show a potent, broad-spectrum antifungal activity^35^. The antifungal protein RsAFP2 from *Raphanus sativus*, a close relative of *B. napus*, has been described to exhibit antifungal activitiy against the fungus *V. dahliae*, which is closely related to *V. longisporum*^36^. Furthermore, a synthetic defensin expressed in *A. thaliana* has also been shown to exhibit antifungal activity against *V. dahliae*^37^. Expansins mediate cell wall-loosening and down-regulation of an expansin-like protein in *A. thaliana* that has been shown to increase resistance against nectotrophic fungi^38^. This suggest that genes affected by PAV and involved in cell wall integrity and signaling at the cell wall surface are key components of *V. longisporum* broad-spectrum resistance in *B. napus*.

We found that in the majority of cases gene presence was associated with resistance against *V. longisporum*. However, interestingly one of the strongest QTL, q23k-BP-C2-3 (R^2^ = 23%) was mapped using SNaP markers only. In this case, the absence alleles were inherited by the parent R53 and associated with resistance. Bionano Optical Mapping confirmed the deletion of a region containing 70 genes in the parent R53. This phenomenon has been rarely reported for quantitative disease resistance, but has previously been shown for Sclerotinia stem rot in *B. napus*^24^ and for three other fungal pathogens in *Medicago truncatula*^39^.

Surprisingly, we found another QTL region containing a total of 7 nucleotide binding site-leucine-rich repeat (NLR) resistance genes (TIR-NBS-LRR) to be affected by PAV and to harbour a QTL on chromosome C09 for *V. longisporum* resistance in the NAM population in this study. This region was also described by Samans *et al*.^18^ to be part of two gene clusters of 14 and 8 TIR-NBS-LRR genes on *B. napus* chromosome C09, which are frequently deleted in natural *B. napus* compared to synthetic *B. napus* accessions. NBS-LRR genes are frequently described to be involved in monogenic disease resistance. For NLR resistance genes it is well known that they are often organized in clusters or tandem repeats in a number of plant species and crops and numerous studies have shown that fitness costs can lead to multiplication and deletion of gene family members, such as in *A. thaliana*^40,41^ and in *B. napus*^17^. *V. longisporum* resistance in the NAM and ExR53-DH population is quantitatively inherited. However, similar co-segregation of TIR-NBS-LRR genes with QTL have been described for other crops, e.g. for soybean^42^, barley^43^, potato^44^ which might result from evolution and local genome diversification of genes involved in qualitative and quantitative disease resistance mechanisms.

The prioritization of these candidate PAV genes mainly involved in cell wall integrity, growth and modification is consistent with our earlier findings that QTL for the concentration of soluble simple phenylpropanoids, which are putative precursors and degradation products of cell-wall modifications and cross-linking, are co-localizing with major resistance QTL in the biparental ExR53-DH population. In addition, the concentrations of some of these cell wall-associated compounds are significantly correlated with *V. longisporum* resistance^45^. Further functional characterization of PAV genes from QTL regions may help to improve our understanding of disease resistance mechanisms and to improve fungal resistance in *B. napus* by exploitation for in future plant breeding programs.

## Materials and Methods

### Phenotyping for *Verticillium longisporum* resistance in *B. napus*

Resistance phenotyping was conducted in the greenhouse at Georg August University Göttingen, Germany. In order to represent a broad range of pathogenicity traits occurring in oilseed rape fields, a spore suspension mixture of *V. longisporum* isolates VL43 (lineage A1/D1, North Germany), VLS3 (lineage A1/D1, Sweden) and PD589 (lineage A1/D3, Japan)^46,47^ with a density of 1 × 10^6^ spores/ml concentrations was used to inoculate the 200 NAM lines applying the root-dipping method^48^ in four experiments. Each experiment included 20 inoculated and 20 control plants for each tested genotype. Rating of symptoms was done weekly over a 4-week period, using the 1–9 disease scoring scale described by Eynck *et al*.^48^. Resistance screening for the biparental population ExR53-DH was performed similarly using a mixture of isolates VL40 and VL43 (both lineage A1/D1) with 202 DH lines as described in detail by Obermeier *et al*.^45^.

### High molecular weight DNA isolation for optical mapping

High molecular weight (HMW) DNA isolation was carried out for Express617 and R53 according to the IrysPrep™ Plant Tissue-Nuclei protocol provided by Bionano Genomics. Young leaves (approximately 2 grams) were harvested from dark-treated rapeseed plants. The harvested leaves were immediately fixed with 2% formaldehyde followed by homogenization in isolation buffer containing PVP-10, BME and Triton X-100. The isolated nuclei were then purified on Percoll cushions. Purified nuclei were further embedded in an agarose matrix. Agarose plugs were further subjected to proteinase K treatment followed by rigorous washings steps. Finally, HMW DNA was recovered by melting the plugs using GELase™ (Epicentre) treatment. An additional drop dialysis step was performed to ensure ultra-clean DNA. High molecular weight DNA was further subjected to sequence-specific nick-labeling using the IrysPrep™ Labeling-NLRS protocol provided by Bionano Genomics. HMW DNA was subjected to digestion by the single-stranded nicking endonuclease *Nt.BspQI* (recognition site GCTCTTC). The nicks created by *Nt.BspQI* were then repaired using fluorophore-labeled nucleotides. Nicked and labeled single DNA molecules were subsequently loaded onto an IrysChip for imaging on the Bionano Genomics Irys system.

### Bio-informatics analysis for Bionano optical mapping data

DNA molecule images generated from the Irys system were computationally translated into single-molecule optical maps. These single molecules were then assembled into consensus maps using the dedicated IrysSolve pipeline (v5134) provided by Bionano Genomics. An *in silico* optical map was generated for the Darmor-*bzh* v. 4.1 ref. ^17^ using Knickers v1.5.5 and was used to calculate noise parameters for the final assembly. Optical map assemblies from Express617 and R53 were finally aligned to the Darmor-*bzh* reference using the runCharacterize script provided by Bionano Genomics, with the settings published by Kawakatsu *et al*.^49^. The alignment was imported into Bionano IrysView (v2.5.1.29842) software for visualizing and detecting structural variations between Express617, R53 and the Darmor-*bzh B. napus* reference genome.

### Global RNA-Seq analysis for confirmation of gene expression

Two contrasting lines from the mapping population ExR53-DH, DH41 (partially resistant genotype) and DH94 (susceptible genotype), were grown for four weeks after inoculation with *V. longisporum*- (isolate VL43) and mock-inoculation in two independent experiments under different environmental conditions (optimal, drought stress and heat stress). For each mock and *V. longisporum*-inoculated plants, pooled hypocotyl samples from 20 plants/line were harvested 28 days after inoculation. In total, 16 samples were analyzed and total RNA was extracted. Sequencing libraries were produced by service provider LGC Genomics GmbH (Berlin, Germany) and in total 1,022 Million 100 bp pair-end raw reads were obtained by Illumina HiSeq 2000 3′end sequencing. Alignment of reads to the *B. napu*s reference genome Darmor-*bzh* v4.1 and all statistical analysis were performed using CLC Genomics Workbench version 9.0 (QIAGEN Bioinformatics CLC bio, Aarhus, Denmark).

### Genetic mapping and QTL analysis

244 DH lines from the F1 of the cross Express617 × R53 and the two parents were analyzed with the Brassica 60 k Illumina Infinium array^26^. DNA was extracted from leaves using the CTAB method^50^ and array genotyping assays were outsourced to TraitGenetics (Seeland, Germany). SNP calls with >85% failed calls across all 244 genotypes were removed from further analyses. Also, SNP probe calls with >90% or <10% of a single allele across the population were removed, leaving a total of 22,064 SNP markers. From these, 4,654 SNP probes (21.1%) showed a segregation pattern with one allele displaying a failed call and 17,410 SNP probes showed a normal biallelic segregation. A “SNaP map” was created from all quality-filtered 22,064 markers (2,714 marker bins), including biallelic and presence/absence polymorphisms, while a “SNP map” was created using only the 17,410 markers showing presence/presence polymorphism (2,176 marker bins). Genetic maps were created using the software MSTMap^51^ applying the kosambi distance function and a cut-off *p* value of 10^−30^. QTL analyses were performed using the software QGene 4.3.9 and 4.4.0^52^ applying composite interval mapping with a scan interval of 1 milliMorgan and automatic forward cofactor selection. Mean normalized AUDPC values from the four *V. longisporum* greenhouse resistance screenings were used as trait input data^45^.

### Genome-wide association studies

The NAM panel was also genotyped using the 60 K Illumina Infinium Brassica SNP array as described above and data was filtered according to Gabur *et al*.^24^. Using the Darmor-*bzh* reference v4.1 we anchored 28,073 SNP markers by BLASTn using CLC Genomics Workbench v. 9.0 (Qiagen Bioinformatics). The SNP map contained 18,068 markers, the SNaP map contained 21,695 markers. Association analyses were conducted using the R package GenABEL^53^. A mixed linear model approach that increases detection power^54^ was adjusted for population stratification by including the kinship matrix and the first two principal components as covariates^55^. Stringent significance cutoff values were set at a false discovery rate (FDR) correction of 10%^56^. To reduce the type II error rate, we also captured the SNP-trait associations for disease resistance using an arbitrary threshold of −log10(p) ≥ 3.

### Linkage disequilibrium (LD) analysis and haplotype construction

Whole genome linkage disequilibrium (LD) was calculated using the squared allele-frequency correlations (r^2^) between pairs of SNPs. Only markers with a maximum of 10% missing data and MAF ≥ 0.05 were included in the analysis. Haplotype patterns were assessed for SNP and SNaP markers that showed significant marker trait association at the adjusted Bonferroni threshold of −log10(P-value) ≥ 4.33. Haplotype blocks were defined with the confidence interval method described by Gabriel *et al*.^57^ in Haploview version 4.2^58^ and visualized with the R package LDheatmap^59^.

### Resequencing and coverage analysis

The Illumina 250 bp paired-end raw sequencing data for the two parents of the biparental population, Express617 and R53, was described previously by Stein *et al*.^60^, while Illumina 100 bp paired-end raw sequencing data for the 6 NAM parents was described by Schmutzer *et al*.^29^. Sequences were aligned to the Darmor-*bzh* v. 4.1 reference using CLC Genomics Workbench v. 9.0 (Qiagen Bioinformatics). Genes from QTL regions were classified as affected or not affected by presence/absence variation based on coverage analysis of the WGS data from the parental lines. Coverage differences were calculated using the bedtools software package v. 2.27.0 with multiBamCov. A minimum cutout threshold of 1.5 aligned reads was used to differentiate between gene presence and absence. The threshold was selected using PCR data for calibration available for the six NAM parental lines from Gabur *et al*.^24^. Additionally, we used the SGSGeneLoss v0.1 described by Golicz *et al*.^30^. The visualization in Fig. 1 was performed using the software MapChart v.2.3, while Fig. 3 was generated using CLC Genomics Workbench 9 track lists. Identification of homoeologous exchanges (HE) was performed using the method described by Samans *et al*.^18^. Further analysis was partly performed by using the R package ‘gsrc’^61^.

### Gene ontology enrichment analyses

To produce gene ontology information for all *B. napus* genes, 101,039 Darmor-*bzh* peptide sequences (Brassica_napus.annotation_v5.pep.fa.gz) were downloaded from the website: http://www.genoscope.cns.fr/brassicanapus/data/ and were used as input for Blast2Go v. 4.1.9. The R package topGO v1.0^62,63^ was used for gene ontology enrichment analysis.

## Supplementary information

Supplementary figures S1, S2 and S3.

Supplementary Tables S1 to S11.
