# Supplementary material for: Gene presence-absence variation associates with quantitative Verticillium longisporum disease resistance in Brassica napus
Source: Sci Rep. 2020 Mar 5;10:4131. doi: 10.1038/s41598-020-61228-3 (PMC7057980; doi:10.1038/s41598-020-61228-3)
Supplement: Supplementary file 1 — Supplementary figures S1, S2 and S3. [file 41598_2020_61228_MOESM1_ESM.pdf]

Scientific Reports, Full Research Paper

Gene presence-absence variation associates with quantitative *Verticillium longisporum* disease resistance in *Brassica napus*

Iulian Gabur[1*], Harmeet Singh Chawla[1*], Daniel Teshome Lopisso[2,3] , Andreas von Tiedemann[2], Rod J. Snowdon[1], Christian Obermeier[1]

[1]Department of Plant Breeding, IFZ Research Centre for Biosystems, Land Use and Nutrition, Justus Liebig University Giessen, 35392 Giessen, Germany
[2]Section of General Plant Pathology and Crop Protection, Georg August University Göttingen, 37077 Göttingen, Germany
[3]College of Agriculture and Veterinary Medicine, Jimma University, Jimma, Ethiopia

[*] These authors contributed equally to this work.

Supplementary Figures: S1 to S3

Supplementary Figure Legends

Supplementary Figure S1. Comparison of genome-anchored 60k Illumina Infinium array polymorphic marker data (a) (4 and 12 replicated samples) with genome-anchored Optical Mapping Bionano data for parental genotypes Express617 and R53 for a 3.4 Mbp section from the end of chromosome C01. Consecutively anchored failed calls (grey boxes) in (a) and missing optical mapping molecule alignment (no blue bars) in (b) indicate long-range deletions (absence) in Express617 or R53.

Supplementary Figure S2. GWAS for *V. longisporum* resistance in NAM population comparing SNP map with SNaP map.

Supplementary Figure S3. Comparison of (a) failed SNaP calls in array genotyping data in red with (b) whole genome sequence coverage data analyses marking deleted regions (red), and homeologous exchanges with duplications in the A genome (light blue) and homeologous exchanges with duplications in the C genome (dark blue) for 6 NAM parental lines.

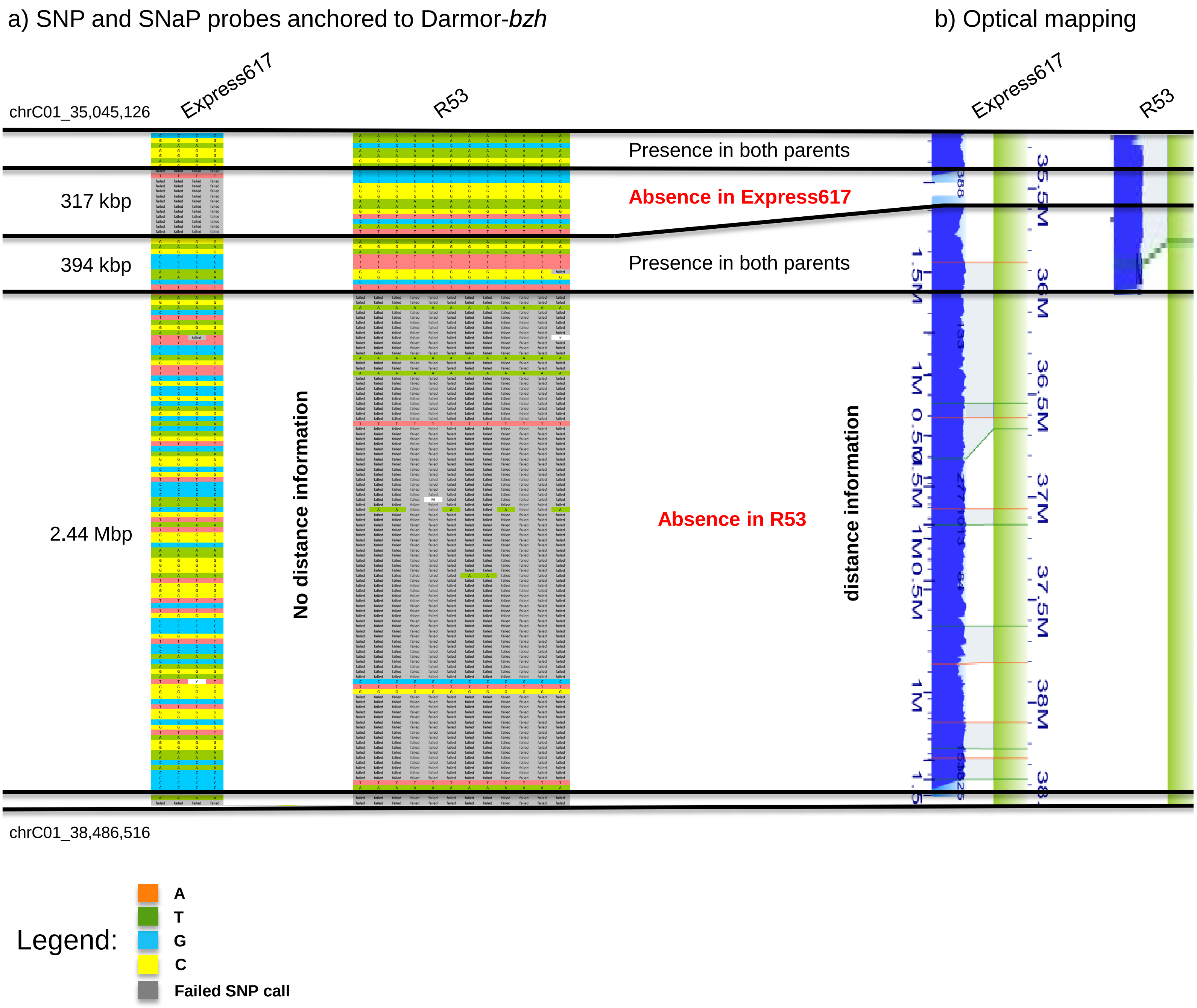
a) SNP and SNaP probes anchored to Darmor-*bzh*
b) Optical mapping
chrC01_35,045,126
Express617
R53
Express617
R53
317 kbp
394 kbp
2.44 Mbp
Presence in both parents
Absence in Express617
Presence in both parents
No distance information
Absence in R53
distance information
35.5M
36M
36.5M
37M
37.5M
38M
1.5M
1M
0.5M
1M
chrC01_38,486,516
Legend:
A
T
G
C
Failed SNP call

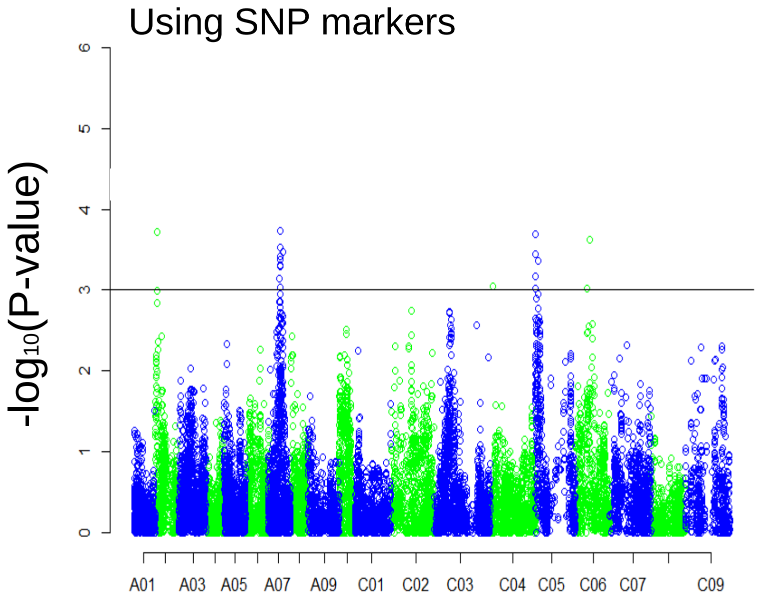
Using SNP markers
-log10(P-value)
0
1
2
3
4
5
6
A01
A03
A05
A07
A09
C01
C02
C03
C04
C05
C06
C07
C09

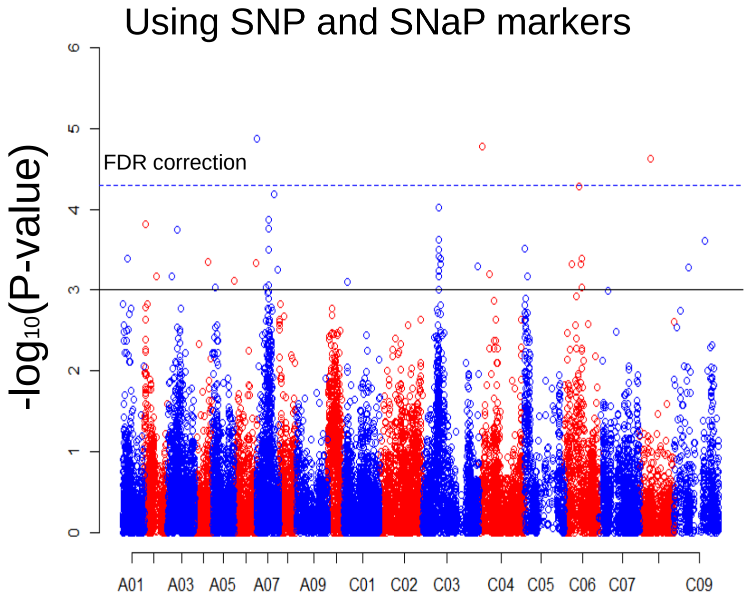
Using SNP and SNaP markers
-log10(P-value)
FDR correction
0
1
2
3
4
5
6
A01
A03
A05
A07
A09
C01
C02
C03
C04
C05
C06
C07
C09

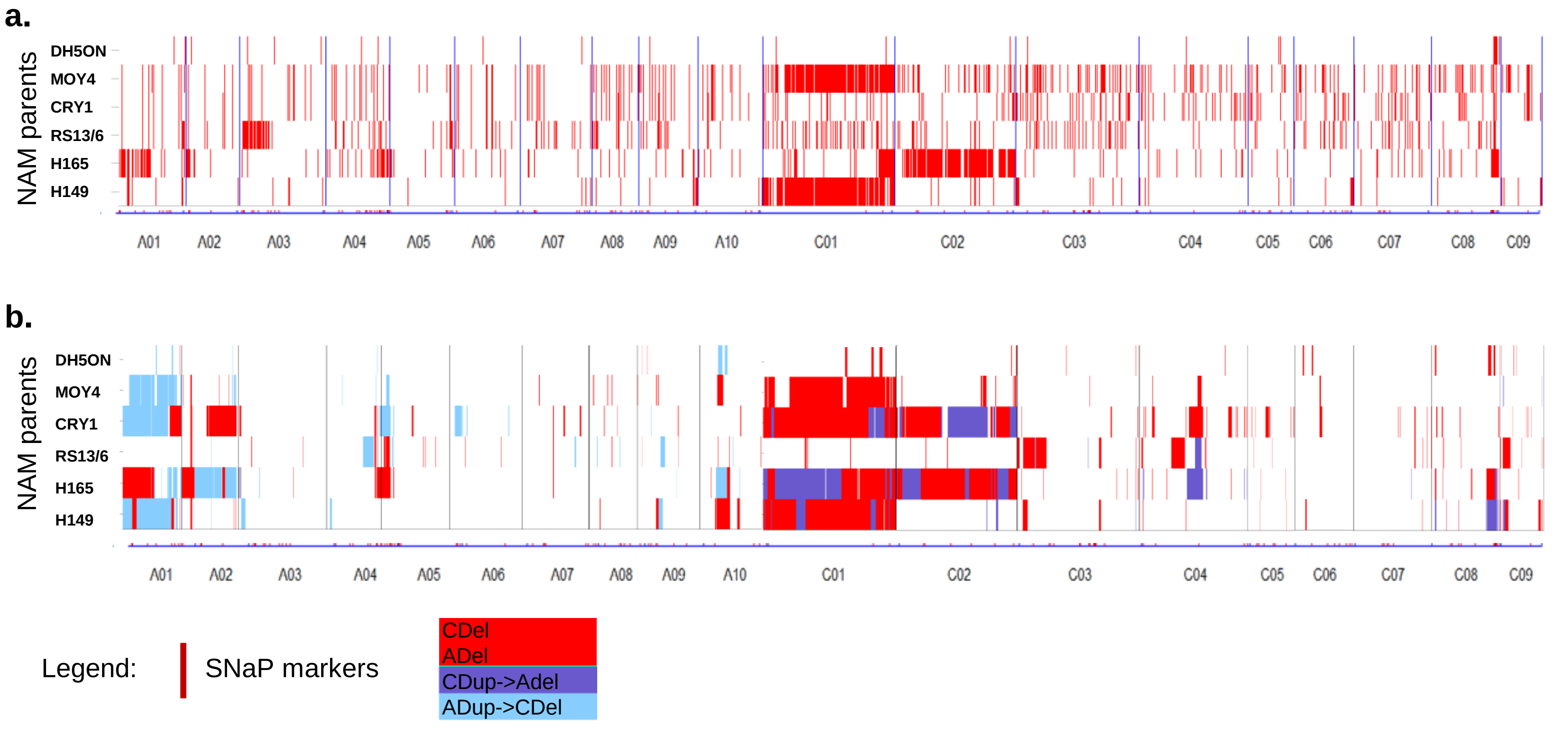
a.
NAM parents
DH5ON
MOY4
CRY1
RS13/6
H165
H149
A01 A02 A03 A04 A05 A06 A07 A08 A09 A10 C01 C02 C03 C04 C05 C06 C07 C08 C09
b.
NAM parents
DH5ON
MOY4
CRY1
RS13/6
H165
H149
A01 A02 A03 A04 A05 A06 A07 A08 A09 A10 C01 C02 C03 C04 C05 C06 C07 C08 C09
Legend:
SNaP markers
CDel
ADel
CDup->Adel
ADup->CDel
